# Editorial: Smart nanomaterials for biosensing and therapy applications

**DOI:** 10.3389/fbioe.2023.1137508

**Published:** 2023-01-17

**Authors:** Xiaofeng Lin, Qitong Huang

**Affiliations:** ^1^ Key Laboratory of Prevention and Treatment of Cardiovascular and Cerebrovascular Diseases of Ministry of Education, Key Laboratory of Biomaterials and Biofabrication in Tissue Engineering of Jiangxi Province, Key Laboratory of Biomedical Sensors of Ganzhou, School of Medical and Information Engineering, Science Research Center, Gannan Medical University, Ganzhou, China; ^2^ Oil-Tea in Medical Healthcare and Functional Product Development Engineering Research Center in Jiangxi, Gannan Medical University, Ganzhou, China

**Keywords:** nanomaterials, biosensing, bioimaging, nanozyme, cancer therapies, triggering immune system

At present, medical science is facing many challenges in early diagnosis and effective treatment of diseases. How to carry out effective diagnosis and treatment of diseases is very important for the improvement of people’s health level (Cao et al., 2022; Diaz et al., 2022; Kathe et al., 2022; Vistain et al., 2022; Xue et al., 2022). Smart nanomaterials have received extensive attention in the field of bioengineering and biotechnology due to their unique structural and functional properties. Compared with traditional materials, smart nanomaterials with special optical, magnetic, electrical and mechanical properties show great potential for applications in biosensing and therapy (Huang et al., 2020; Liu et al., 2022a; Zhang et al., 2022a; Yi et al., 2022). The successful development of the smart nanomaterials has greatly improved the accuracy of disease diagnosis and treatment efficiency.

To present state-of-the-art research in this field, we launched a Research Topic in Frontiers in Bioengineering and Biotechnology entitled “*Smart Nanomaterials for Biosensing and Therapy Applications*.” A total of 20 articles have been published in this Research Topic, including 11 original research articles, 7 review articles and 2 mini review articles, which represents the recent advances in investigations on biosensing, bioimaging, nanozyme, cancer therapies and triggering immune system.

As known, biosensor is an analytical instrument composed of biological-receptors, transducers and signal-processors. The main principle of the biosensor is to combine biometric elements with sensors to produce a measurable signal proportional to the concentration of the analyte, which can be used to detect, transmit and record the physical, chemical and biological reactions of the analytes. Over the past few decades, biosensors have been used to replace traditional analytical techniques because they are reusable, specific, sensitive, and allow for rapid and multiple assays. Therefore, the biosensors have been widely used in disease diagnosis, drug analysis, environmental monitoring, food safety and other fields (Lin et al., 2021; Mei et al., 2022a; Huang Q et al., 2022). Due to the smart nanomaterials have good catalytic, conductive and optical properties, they can greatly improve the detection performance of biosensors.

Liu et al. constructed a fiber-based triboelectric nanogenerators with good stability, washability, flexibility and stretchability, which can be attached to the human body to monitor the finger, arm, and leg activities. What’s more, the fiber-based triboelectric nanogenerators can be used as the power supply of the temperature sensor in a self-powered sensing-system. This work is expected to be applied in multi-functional motion-sensors and human health monitoring.

The sensitive and accurate analysis of circulating tumor cells (CTCs) plays an important role in the early diagnosis and prognosis of cancer patients. Li et al. reviewed the application of microfluidics in detection of CTCs and the potential clinical application of CTCs. At the same time, the application prospect of microfluidics in the treatment of tumor metastasis had been prospected, the development prospect of microfluidic-biosensors had been also discussed.

Electrochemical biosensors have been applied in many fields because of their advantages of economy, environmental protection, simplicity and high sensitivity. Mei et al. summarized the detection of SARS-CoV-2 based on electrochemical DNA biosensors. The development trend of the electrochemical DNA biosensors for SARS-CoV-2 detection is prospected, which can provide reference for researchers in the establishment of different detection methods. Zhang et al. reviewed and discussed the basic principle, nanomaterials, actuality and future development trend of electrochemical biosensors for the detection of lipid hormone.

How to achieve effective treatment and prevention of disease is one of the key factors related to human health and enhance human wellbeing. With the development of science and technology, smart nanomaterials have been widely used in the treatment and prevention of diseases because of their advantages in biocompatibility, targeted-therapy, drug-delivery, photothermal-therapy, etc.

Li et al. reviewed the advances in nanomaterials for immunotherapy, including nanoparticle-based delivery systems, nanoparticle-based photothermal and photodynamic immunotherapy, nanovaccines, nanoparticle-based T Cell cancer immunotherapy and photodynamic immunotherapy, and nanoparticle-based bacterial cancer immunotherapy. Zhu et al. used self-emulsifying nanotechnology to link iodized oil and indocyanine green (ICG), then used Tween-80 and lecithin to emulsify the mixture of iodized oil and ICG to produce a new type of smart nanomaterials. Comparing with the free ICG, the fluorescence intensity of the prepared smart nano-composite is obviously enhanced, and the fluorescence stability and photobleaching resistance of the nanoemulsion were greatly improved under the shielding action of iodol, and the nanoemulsion had the ability of long-term continuous real-time *in vivo* imaging. In addition, the smart nanomaterial can successfully localize tumor tissue and has good tumor-to-normal contrast. Under the fluorescence guidance of the smart nanomaterial, the tumor tissues of orthotopic liver cancer mice can be clearly visualized and accurately delineated, which can achieve effective resection.

It is well-known that the level of reactive oxygen species (ROS) in the body is closely related to the related diseases of the human body. The imbalance of ROS secretion can cause cancer, dry eye disease, cardiovascular disease and so on. Therefore, it is possible to achieve the treatment of related diseases by adjusting the level of ROS in the body with appropriate therapeutic methods. Li et al. synthesized a viable and efficient tantalum-carbon nanozyme with excellent catalase-like (CAT) activity and radiosensitivity by *in situ* reduction of an ultra-small tantalum nanase in a metal-organic framework material (MOF)-derived carbon nanase. The above smart nanomaterials can significantly improve the CAT activity of carbon nanomases and promote the production of more ROS. By improving ROS levels, more DNA double-strand breaks were present at the cellular level, which in turn increased radiotherapy sensitivity, resulting in the excellent antitumor activity of this smart nanomaterial (Li et al.). Zha et al. successfully encapsulated the NLRP3 inhibitor in polydopamine-based microgel for the treatment of dry eye. The results showed that the smart nanomaterial had good biocompatibility, which could prolong the retention time of the drug on the ocular surface, and could effectively inhibit the corneal epithelial injury and apoptosis. In addition, due to the synergistic effects, the NLRP3 inhibitor-loaded microgels exert stronger ROS clearance at lower doses to suppress inflammation. Li et al., summarized a variety of metal-based nanozymes that regulate ROS levels in the body to achieve treatment of related inflammatory diseases, they also discussed the potential applications of metal-based nanozymes as biomedical materials. Li et al. fixed superoxide dismutase (SOD) in zeolite imidazolate framework-8 (SOD@ZIF-8) by biomimetic mineralization method to treat nerve injury by eliminating ROS . The ROS scavenging activity of SOD@ZIF-8 plays a key role in protecting SHSY-5Y cells from MPP + induced cell model damage and alleviating apoptosis, suggesting that SOD@ZIF-8 can effectively treat ROS-mediated nerve damage by removing excess ROS produced *in vitro*.

In order to improve the therapeutic effect of tumors, many therapeutic methods have been developed. Photodynamic therapy (PDT) and photothermal therapy (PTT) have been widely used in the treatment of many solid tumors in recent years due to its advantages of light-triggered, less trauma and high space-time selectivity. Chen et al. summarized the advantages and disadvantages of PDT based on metal nanomaterials, providing a reference for the application of nanomaterials in PDT therapy. Li et al. prepared Cu_2_MoS_4_@MXene smart nanocomposite, which has obvious photothermal conversion efficiency (*η* = 87.98%). Based on the electron transfer regulation effect and local surface plasmon resonance effect, a mechanism is proposed to promote non-radiation recombination and generate more heat, and the material has a very good effect in the treatment of cancer. The synthesis method, photothermal mechanism and cancer treatment scheme of the above smart nanomaterial can be extended to other photothermal agents to achieve more effective treatment of cancer.

Because of the excellent biological properties of smart nanomaterials, their applications in drug delivery are getting more and more attention. Yang et al. developed an exosome-loaded camptothecin nano-drug delivery platform as a personalized treatment modality for the treatment of cervical cancer. This method can significantly improve the sensitivity of tumors to ionizing radiation, which can improve the overall efficacy of radiotherapy without the need for higher radiation doses. Chen et al. developed a self-powered drug delivery system consisting of a disk TENG (D-Teng), a rectifying bridge and two gold electrodes. The D-Teng has good performance and provides long-term stable current. The output current of the D-Teng is converted to direct current by the rectifier bridge to stimulate the cells/tissues between the gold electrodes. *In vitro* experiments showed that the system can significantly improve the utilization of doxorubicin. Wang et al. summarized the opportunities and challenges of biomimetic exosomes in drug delivery systems over the past few decades. The design, synthesis, and biomedical applications of stimulus-responsive polymeric prodrugs of paclitaxel are reviewed by Zhou et al., they also summarized the opportunities and challenges of the field. Tian et al. reviewed the application of hydrogels in osteosarcoma inhibition and bone regeneration, and made some suggestions for future development.

Aluminum adjuvants have been widely used in some vaccines and can induce strong human immunity. However, deficiencies in cellular immunity limit its use in some vaccines. Therefore, there is an urgent need to develop novel adjuvants to enhance humoral and cellular immune responses. Shan et al. designed and prepared a novel nano adjuvant (PF3) by microfluidization by combining saponins (ginsenoside Rg1) with oil-in-water nanoemulsion (NE). The PF3 has a stronger humoral and cellular immune induction effect than aluminum adjuvants because of its higher cellular uptake and ability to activate immune response pathways. In addition, the size and zeta potential of PF3 did not change significantly after being stored at 4°C and 37°C for 12 weeks, indicating high stability *in vitro*. This study provides an adjuvant platform for the design of recombinant vaccines instead of traditional aluminum adjuvants.

Zika virus (ZIKV) seriously affects the normal development of the fetus. Visualizing the pathogenic activity of ZIKV from the route of infection to the immune process is critical. However, accurate labeling of ZIKV remains challenging due to the lack of reliable labeling techniques. Zheng et al. designed a new fluorescent quantum dots (QD)-based probe for reliable labeling and visualization of ZIKV. The probe conjugates ZIKV and QD with high efficiency by photo-activated bio-orthogonal cycloaddition. The labeling process is simple and has no effect on the permeability. The results show that the combination of ZIKV and QD probe can localize and track the function of the virus during infection. Therefore, this bio-orthogonal QD probe may be a promising method for detecting the pathogenic activity of ZIKV.

Mesoporous dopamine nanoparticles (MPDA NPs) are a kind of antitumor nanomaterials with broad clinical application prospects, but their biosecurity remains unclear. Huang et al. analyzed the toxicity of RNA-Seq-based MPDA NPs in mice following different exposure routes. The results showed that RNA-Seq-based MPDA NPs of different administration routes had no obvious or serious toxic effect on mice. The authors elucidate the toxic effect of RNA-Seq-based MPDA NPs from the molecular mechanism, which provides a new insight for the further clinical application of MPDA NPs.

Smart nanomaterials have a wide range of applications. The contributions in this Research Topic provide a variety of smart nanomaterials and their applications in biosensing and therapeutics, including biosensors, bioimaging, nanozyme, cancer therapies and triggering immune system. We believe that this Research Topic will provide new ideas and technologies for the application of smart nanomaterials in biosensing and therapy. More importantly, in order to get the latest achievements in this field, the Research Topic has launched the second Research Topic, entitled “*Smart Nanomaterials for Biosensing and Therapy Applications* II,” welcome to submit!
